# The Virus of Influenza

**Published:** 1923-03

**Authors:** 


### THE VIRUS OF INFLUENZA.

MUCH more authentic information must be received before we can accept as a fact the recently reported American discovery of the cause of influenza. Possibly the question has at last been settled, but meanwhile it is well to be quite clear exactly where we stand with regard to the bacteriology of this disease. After several years of research bacteriologists have become divided into two groups, those who considered the bacillus originally discovered by Pfeiffer to be the true infecting agent, and those who, as a cause, claim a minute ultra-microscopic virus so small that it passes through the finest filters. Recently two particularly valuable additions to our knowledge have been published, and they have tended so satisfactorily to reconcile the two opposing theories that one cannot help feeling that the real "discovery" had probably been made before the latest news from America arrived. Working in Denmark, Kristensen published in the latter part of 1922 a remarkably complete study of Pfeiffer's bacillus, and appears almost to have proved that this organism is not the cause of influenza as a disease, but simply thrives in the respiratory passages of catarrhal patients, particularly those who are actually suffering from influenza.

CONSEQUENCE, NOT CAUSE.

Influenza comes first, the Pfeiffer's bacillus arrives later. Throughout an influenza epidemic it flourishes secondarily in many throats, and so it came to be considered as the cause itself. Actually its arrival is simply the consequence. On the other hand Sir Spencer Lister in South Africa has recently repeated the isolation of a filter-passing virus for actual cases of influenza, but has also carried the investigation a stage further by obtaining a number of human volunteers upon whom to attempt experimental infection with the germ. Cultures were gently sprayed into the noses and throats of the volunteers, and three of them developed a rise of temperature, and the symptoms of uncomplicated influenza. The hypercritical will say that even this experiment does not finally prove the case for the filter passer, but it seems to go far towards doing so. It appears as though the latest announcement simply implies a further study in the life history of this filter passing germ, and its value should therefore be viewed in relation to work already done, as a link in the chain of systematic research and not as a dramatic and far-reaching "discovery."

